# IL-1β contributes to the secretion of sclerostin by osteocytes and targeting sclerostin promotes spinal fusion at early stages

**DOI:** 10.1186/s13018-023-03657-0

**Published:** 2023-03-03

**Authors:** Zengxin Jiang, Lixia Jin, Chang Jiang, Zuoqin Yan, Yuanwu Cao

**Affiliations:** 1 Department of Orthopaedics, Shanghai Sixth People’s Hospital, Shanghai, 200233 China; 2 Department of Orthopedic Surgery, Zhongshan Hospital, Fudan University, NO. 180 Feng Lin Road, Xuhui District, Shanghai, 200032 China

**Keywords:** Sclerostin, Spinal fusion, Osteocyte, Osteoblast, IL-1β

## Abstract

**Background:**

Despite extensive research, there is still a need for safe and effective agents to promote spinal fusion. Interleukin (IL)-1β is an important factor which influences the bone repair and remodelling. The purpose of our study was to determine the effect of IL-1β on sclerostin in osteocytes and to explore whether inhibiting the secretion of sclerostin from osteocytes can promote spinal fusion at early stages.

**Methods:**

Small-interfering RNA was used to suppress the secretion of sclerostin in Ocy454 cells. MC3T3-E1 cells were cocultured with Ocy454 cells. Osteogenic differentiation and mineralisation of MC3T3-E1 cells were evaluated in vitro. SOST knock-out rat generated using the CRISPR-Cas9 system and rat spinal fusion model was used in vivo. The degree of spinal fusion was assessed by manual palpation, radiographic analysis and histological analysis at 2 and 4 weeks.

**Results:**

We found that IL-1β level had a positive association with sclerostin level in vivo. IL-1β promoted the expression and secretion of sclerostin in Ocy454 cells in vitro. Inhibition of IL-1β-induced secretion of sclerostin from Ocy454 cells could promote the osteogenic differentiation and mineralisation of cocultured MC3T3-E1 cells in vitro. The extent of spinal graft fusion was greater in SOST-knockout rats than in wild-type rats at 2 and 4 weeks.

**Conclusions:**

The results demonstrate that IL-1β contributes to a rise in the level of sclerostin at early stages of bone healing. Suppressing sclerostin may be an important therapeutic target capable of promoting spinal fusion at early stages.

**Supplementary Information:**

The online version contains supplementary material available at 10.1186/s13018-023-03657-0.

## Introduction

Spinal fusion is one of the most commonly used operations to treat spinal instability and spinal degenerative diseases. The number of lumbar spinal fusion procedures performed in the United States between 2004 and 2015 has increased by 62.3% and the costs also increased [1]. Autologous iliac crest bone graft is the “gold standard” of spinal fusion. But even with autologous graft. But even with autologous graft, the efficacy of spinal fusion is not always satisfactory [2, 3].

A number of osteogenic agents that stimulate bone growth have been identified. Bone morphogenetic protein (BMP) is a growth factor necessary for bone tissue development and repair [4, 5]. Bone morphogenetic protein-2 (BMP-2) has been approved by the US Food and Drug Administration (FDA) for anterior lumbar fusion in the spine, but safety concerns including postoperative associated adverse effects, ectopic bone formation, osteoclast-mediated bone resorption and inappropriate adipogenesis have emerged [6–9]. Small molecules that can stimulate the differentiation of human bone marrow-derived mesenchymal stromal cells (hBMSCs) into osteoblasts or enhance the effects of BMP2 on hBMSCs have been developed, but there is currently no evidence that these small molecules promote bone growth in vivo [10–13]. Therefore, it is necessary to identify safe and effective osteogenic agents that promote spinal fusion.

Sclerostin, which is encoded by the *SOST* gene, is a negative regulator of bone formation. Sclerostin is primarily secreted by mature osteocytes. Sclerostin is retained in the bone compartment by binding to the low-density lipoprotein receptor-related protein (LRP4) receptor located on the osteoblast membrane. Sclerostin also binds to LRP5/6 and inhibits canonical Wnt signalling, thereby exerting its negative regulatory effects [14, 15]. The negative regulatory effects of sclerostin include inhibition of canonical Wnt signalling to suppress the proliferation and differentiation of osteoblasts/pre-osteogenic cells; suppression of mature osteoblast activity; promotion of osteoblast apoptosis; and inhibition of bone mineralisation [16–18]. Studies have shown sclerostin increase dramatically in the early stages of the healing process, which may have potential problems for the development of successful spinal fusion [19, 20]. But the factors fueling this rise are not fully known.

Inflammation is an important factor affecting spinal fusion. Similar to the fracture healing process, bone graft healing involves an early stage of inflammation, in which inflammatory cytokines such interleukin-1β (IL-1β) and tumour necrosis factor-α (TNFα) are attracted to the bone graft site and help regulate the repair and resorption (i.e. remodelling) of skeletal tissues [21–23]. Increasing evidence supports the notion of IL-1β-mediated bone loss [24, 25]. IL-1β has also been proven to inhibit osteogenic differentiation of mesenchymal stem cells and induce cell apoptosis, resulting in decreased bone volume and mechanical strength and ultimately impairing bone healing [26]. Therefore, the influence of inflammation on spinal fusion should not be ignored.

Growing evidences have shown that IL-1β is significantly increased at early stages of bone healing, which is similar to sclerostin [27, 28]. But it is unclear whether IL-1β is a cause of increased sclerostin. In addition, considering the proven efficacy of romosozumab (an anti-sclerostin antibody) in osteoporosis treatment, anti-sclerostin treatment may effectively promote the healing of the vertebrae [29, 30]. But sufficient evidence is still lacking [31]. Therefore, we conducted the current experiment to determine the connection between IL-1β and sclerostin in osteocytes and to explore whether inhibiting the secretion of sclerostin from osteocytes promotes spinal fusion at early stages.

## Materials and methods

### Cell culture and coculture experiments

Ocy454 cells, a murine osteocyte line, were kindly provided by Prof. Paola Divieti Pajevic (Boston University, Henry M. Goldman School of Dental Medicine). MC3T3-E1 cells were purchased from The Cell Bank of Type Culture Collection of the Chinese Academy of Sciences. Ocy454 cells were differentiated in α-minimum essential medium (α-MEM, Gibco; Thermo Fisher Scientific, Inc.) containing 10% fetal bovine serum (Gibco) at 37 °C for 10 days. Cells were then trypsinised and subcultured in 6-well plates (2 × 10^5^ cells per well) or 96-well plates (1 × 10^4^ cells per well) for 12 h before subsequent experiments. For coculture experiments, Ocy454 cells were seeded onto 0.4-mm-thick polycarbonate inserts (Costar Corning, Life Sciences, Acton, MA, USA) in 24-well plates while MC3T3-E1 cells were plated in 24-well plates in α-MEM medium. Ocy454 and MC3T3-E1 cells were cocultured in a mixed medium (α-MEM and osteogenic differentiation medium, 1:1 ratio; Cyagen) for 28 days. The medium was replaced every 2 days, and the inserts seeded with Ocy454 cells were replaced every 4 days to prevent unwanted effects associated with excessive cell density.

### Cell viability

Cell viability was assessed using a Cell Counting Kit-8 (CCK-8) assay (Beyotime Institute of Biotechnology, Nanjing, China). Briefly, Ocy456 and MC3T3-E1 cells were seeded in 96-well plates (1 × 10^4^ cells per well). Following exposure to different concentrations of IL-1β (0, 1, 5, 10, or 20 ng/ml) for 24 h, the cells were incubated with 100 µl of serum-free α-MEM containing 10% CCK-8 solution for 2 h at 37 °C. The absorbance at 450 nm was measured using a microplate reader (Epoch; BioTek Instruments, Inc.).

### Small-interfering RNA (siRNA) and cell transfection

Three siRNA sequencies specifically targeting *SOST* were designed and synthesised by RiboBio (RiboBio Co. Ltd., Guangzhou, Guangdong, China): 5ʹ-CTGAGAACAACCAGACCAT-3ʹ (siRNA1), 5ʹ-ATCCCTATGACGCCAAAGA-3ʹ (siRNA2) and 5ʹ-ACACCCGCTTCCTGACAGA-3ʹ (siRNA3). *SOST*-siRNA was transfected into Ocy454 cells using Lipofectamine 2000 (Thermo Fisher Scientific, Inc.). After 48 h of culture, the cells were collected for future experiments.

### Enzyme-linked immunosorbent assay (ELISA)

The level of sclerostin secreted by Ocy454 cells and the levels of IL-1β and sclerostin in rat bone tissues of transverse process were measured using a sclerostin ELISA kit (SU-B31033, Kenuodi, Quanzhou, China) and a IL-1β ELISA kit (SU-B30419, Kenuodi, Quanzhou, China) according to the manufacturer’s instructions. Transverse processes of at L4 and L5 in rats were decorticated using a drill. Decorticated bone tissues were harvested at day 1, 3, 7, 14 and 28. Transverse processes of L4 and L5 in unoperated rats were used as control (day 0). Equal weight of bone tissue was repeatedly crushed in 5 ml PBS and the supernatant was collected as previously reported [32, 33]. The standard samples, experimental samples and antibody were added to a microplate. After three washes, the 3,3′,5,5′-tetramethylbenzidine colourimetric reagent and the stop solution were added to the wells in succession. The absorbance at 450 nm was measured using a microplate reader. The concentrations of IL-1β and sclerostin were calculated from standard curves.

### Alkaline phosphatase (ALP) staining and activity measurement

ALP was stained using a BCIP/NBT alkaline phosphatase color kit (C3206, Beyotime). After 7 days osteogenic induction, cells were fixed with 4% paraformaldehyde and subjected to ALP staining according to manufacturer’s instructions. The ALP activity of MC3T3-E1 cells was measured using an alkaline phosphatase assay kit (P0321S, Beyotime). The cells were lysed using radioimmunoprecipitation (RIPA) lysis buffer and centrifuged at 3000 rpm for 20 min. The supernatant was then transferred to a 96-well plate and incubated at 37 °C for 30 min in the presence of *p-*nitrophenol and the reaction substrates. ALP activity was determined after measuring the absorbance of the *p*-nitrophenyl substrate at 405 nm on a microplate reader.

### Real-time polymerase chain reaction (PCR) assay

After 7 days of coculture, total RNA was extracted from MC3T3-E1 cells using TRIzol reagent. The concentration and quality of the extracted RNA were measured by a Nanodrop spectrophotometer (DeNovix, Wilmington, DE, USA). cDNA was synthesised from 1 µg of RNA using Reverse Transcript Master Mix (Thermo Fisher Scientific, Inc.). Quantitative PCR amplification was performed using an Applied Biosystems QuantStudio 5 Real-Time PCR System with qPCR Master Mix Kit and the following thermocycling conditions: melting at 95 °C for 10 s, annealing at 95 °C for 5 s and extension at 60 °C for 20 s for 45 cycles. The cycle threshold values were normalised for the expression of glyceraldehyde 3-phosphate dehydrogenase (GAPDH). The forward and reverse primer sequences used are listed in Table 1.

**Table 1:** Primers’ Sequences Used in the Real-Time PCR (5'—3')

Name	Forward	Reverse
GAPDH	AAGGTCGGTGTGAACGGATT	TGAGTGGAGTCATACTGGAACAT
Sclerositn	CGTGCCTCATCTGCCTACTTGT	CGGTTCATGGTCTGGTTGTTCT
ALP	ACGGCGTCCATGAGCAGAACTA	CAGGCACAGTGGTCAAGGTTGG
OCN	CCAAGCAGGAGGGCAATAAGGT	CTCGTCACAAGCAGGGTTAAGC
OPN	AGCAAGAAACTCTTCCAAGCAA	GTGAGATTCGTCAGATTCATCCG
Runx2	CCCAGGCAGTTCCCAAGCATTT	GGTAGTGAGTGGTGGCGGACAT

### Western blot assay

The total protein fraction was extracted from MC3T3-E1 cells (7 days after coculture) and Ocy454 cells (48 h after transfection) using RIPA assay buffer (Beyotime). The protein concentrations were measured using a bicinchoninic acid protein assay kit (Thermo Fisher Scientific, Inc.). Total protein (20 µg per sample) was separated by sodium dodecyl sulphate–polyacrylamide gel electrophoresis on a 12% gel and then transferred to a nitrocellulose membrane (EMD Millipore). The membranes were incubated overnight at 4 °C with the following antibodies: ALP (1:1,000; ab229126, Abcam, Cambridge, MA, United States), osteocalcin (OCN; 1:1,000; 23,418–1-AP, Proteintech, Wuhan, China), osteopontin (OPN; 22,952-AP, 1:1,000; Proteintech), Runt-related transcription factor 2 (Runx2; 1:1,000; #12,556, Cell Signaling Technology, Danvers, MA, United States), sclerostin (1:1,000; ab85799, Abcam) and β-actin (1:2,000; #4970, Cell Signaling Technology). Membranes were washed with Tris-buffered saline containing Tween (0.05%) for 30 min, followed by incubation with anti-rabbit secondary antibody (1:5,000; Cell Signaling Technology) for 2 h at room temperature. The protein signals were visualised using an Enhanced Chemiluminescence Plus kit (Tanon Science and Technology Co., Ltd.). Densitometry analysis was performed using ImageJ software (version 1.8.0; National Institutes of Health, Bethesda, MA, USA).

### Alizarin red staining

After 21 days of coculture, the MC3T3-E1 cells were gently washed with phosphate-buffered saline (PBS) twice and fixed in 4% paraformaldehyde solution for 20 min. The cells were then washed with PBS twice to remove residual paraformaldehyde solution. The cells were stained with Alizarin red S staining solution for 30 min, washed with PBS and dried naturally. Five images were taken of each sample using an Olympus fluorescence microscope at a magnification of 40 × . The mineralised modules were quantified using Image J software.

## ***Production of SOST***^−/−^*** rats***

Animal study was approved by the Ethics Committee of Fudan University Zhongshan Hospital. *SOST*-knockout (*SOST*^−/−^) Sprague–Dawley (SD) rats were established by Jiangsu GemPharmatech Co., Ltd (Nanjing, China) using the CRISPR-Cas9 system. Transcript SOST-201 (ENSRNOT00000028238.2) was selected to produce SOST − / − rats. SOST-201 gene has 2 exons, with the ATG start codon in exon1 and TAG codon in exton2. Cas9 mRNA and sgRNA were co-injected into zygotes. sgRNA direct Cas9 endonuclease cleavage in intron 1–2 and downstream of exton2, and created a double-strand break. Such breaks would be repaired by non-homologous end joining and result in disruption of SOST gene. The pups were genotyped by PCR, followed by sequence analysis. The sequences of genes and sgRNAs were provided in the Additional file 1.

### Animal models

12 male *SOST*^−/−^ rats and 12 male SD rats (wild-type, WT, obtained from Shanghai SLAC Laboratory Animal Company, Shanghai, China) were used in the current study. All rats were used at the age of 12 weeks. Lumbar spine bone mineral density (BMD), femur BMD and total BMD (skull excluded) of rats were measured with dual X-ray absorptiometry (iNSiGHT VET DXA; OsteoSys, Seoul, Korea) for bone phenotype assessment. The rats were then divided into four experimental groups (n = 6 per group): 2-week WT rats, 2-week *SOST*^−/−^ rats, 4-week WT rats and 4-week *SOST*^−/−^ rats. Rats were anesthetized using sodium pentobarbital. Bilateral posterolateral intertransverse process lumbar fusion surgery was performed using autologous iliac crests. The L4 and L5 levels were identified with reference to the iliac crests. A single midline skin incision and two paramedian fascial incisions were made to expose the transverse processes of L4 and L5. The fusion site was decorticated using a drill. The autologous iliac grafts (a quarter of each ilium from each rat) were then placed over the bed space (L4 to L5) on both sides of the spine. After surgery, the rats were monitored to detect neurological symptoms or infections. The rats were sacrificed at 2 or 4 weeks after surgery, according to the allocated group.

### Manual palpation

The soft tissues were carefully removed from around the spine, and three observers (blinded to the experimental groups) manually palpated the spine, and scored it as follows. Compared with adjacent joints, an operated joint with no reduction in motion was scored as 0. An operated joint with reduced mobility, indicating partial fusion, was scored as 1. An operated joint that was immobile, indicating complete fusion, was scored as 2 [34].

### Radiographic analysis

Micro-computed tomography (micoCT) scans of the spines were preformed using SCANCO 50 (Switzerland). Three-dimensional reconstructed images of the fused spine segment were generated. The CT images were evaluated by three radiologists (blinded to the experimental groups) and scored as follows for each side of the fusion mass. Robust fusion between L4 and L5 was scored as 2. A continuous, but narrow fusion mass, indicating partial fusion, was scored as 1. A discontinuous fusion mass, indicating no fusion, was scored as 0 [34]. Fusion-mass volume on microCT was calculated using ImageJ software and the Volumest (VOLUMe ESTimation) plugin.

### Histological analysis

Rat spines were harvested and fixed with 4% paraformaldehyde overnight. The spines were decalcified in 10% ethylenediaminetetraacetic acid (EDTA) for 4 weeks and embedded in paraffin. The paraffin-embedded tissues were cut into 4 μm paraffin sections. Hematoxylin and eosin (H&E) staining and Masson trichrome staining were conducted to evaluate the formation of bone according to standard histological protocols as described previously [35, 36]. The slides were scanned using an Olympus BX51 microscope (Olympus, Tokyo, Japan).

### Statistical analysis

In vitro experiments were independently carried out three times. Data are presented as the mean ± standard deviation. Statistical comparisons among multiple groups were performed using one-way analysis of variance (ANOVA) followed by Bonferroni’s multiple comparison test or a Kruskal–Wallis test (nonparametric, manual palpation score and micro-CT fusion score). Two group comparisons were done using Student’s t-test. The correlation analyses were performed using Pearson correlation. *p* values of < 0.05 were considered statistically significant. All statistical analyses were performed using SPSS software (version 22.0; IBM Corp.).

## Results

### IL-1β promoted the expression and secretion of sclerostin from Ocy454 cells in vitro

**Fig. 1:**
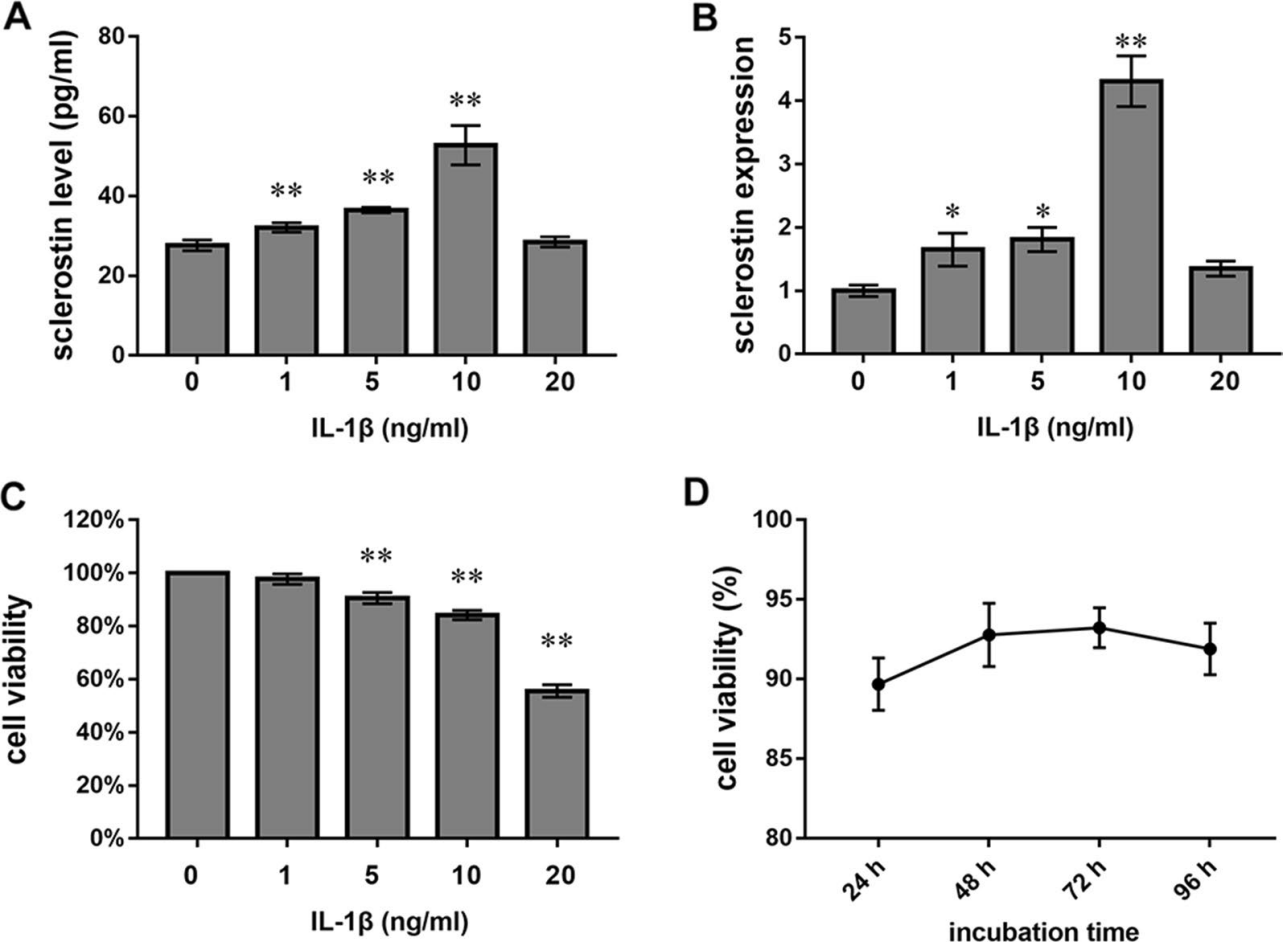
Effects of IL-1β on the viability of Ocy454 and MC3T3-E1 cells and secretion of from Ocy454 cells. **A**, **B** Effects of exposure to different concentrations of IL-1β (0, 1, 5, 10, or 20 ng/ml) for 24 h on the secretion of sclerostin (**A**) and sclerostin mRNA expression levels (**B**) in Ocy454 cells. **C** Effects of exposure to IL-1β (0, 1, 5, 10, or 20 ng/ml) for 24 h on the viability of Ocy454 cells. **D** Effects of exposure to 10 ng/ml IL-1β for 24, 48, 72, or 96 h on the viability of MC3T3-E1 cells. The experiments in vitro were performed independently 3 times. *p < 0.05 and **p < 0.01 versus control cells (0 ng/ml IL-1β)

We performed qPCR and ELISAs to measure sclerostin mRNA expression and secretion from Ocy454 cells. A CCK8 assay was used to determine the viability of Ocy454 and MC3T3-E1 cells. We found that exposure to 1, 5 or 10 ng/ml IL-1β significantly increased sclerostin mRNA expression and secretion from Ocy454 cells (Fig. 1A, B). Exposure to 1 ng/ml IL-1β did not affect the viability of Ocy454 cells. Although 5 or 10 ng/ml IL-1β decreased the viability of Ocy454 cells, the viability remained above 80% (Fig. 1C). Exposure to 20 ng/ml IL-1β did not significantly affect sclerostin mRNA expression or secretion compared with those in the control group. This finding may be explained by the marked decrease in cell viability (around 50%). Based on these results, we used IL-1β at a concentration of 10 ng/ml in subsequent experiments. Considering the subsequent coculture experiments, we also determined the effect of 10 ng/ml IL-1β on the viability of MC3T3-E1 cells. In this experiment, the viability of MC3T3-E1 cells remained above 85% after incubation for 24, 48, 72 and 96 h (Fig. 1D).

### SOST-siRNA transfection inhibited sclerostin expression and secretion of Ocy454 cells

*SOST*-siRNA was transfected into Ocy454 cells and was confirmed to inhibit sclerostin expression and secretion, as determined by PCR and western blotting. Of the three siRNAs tested, *SOST*-siRNA2 elicited the greatest inhibitory effects on sclerostin mRNA and protein expression (Additional file 2: Fig. S1A–C). Furthermore, *SOST*-siRNA2 transfection significantly decreased the secretion of sclerostin from Ocy454 cells (Additional file 2: Fig. S1D). Thus, siRNA2 was used in following experiments, unless otherwise specified.

### Inhibiting the secretion of sclerostin from Ocy454 cells promoted osteogenic differentiation of MC3T3-E1 cells

**Fig. 2:**
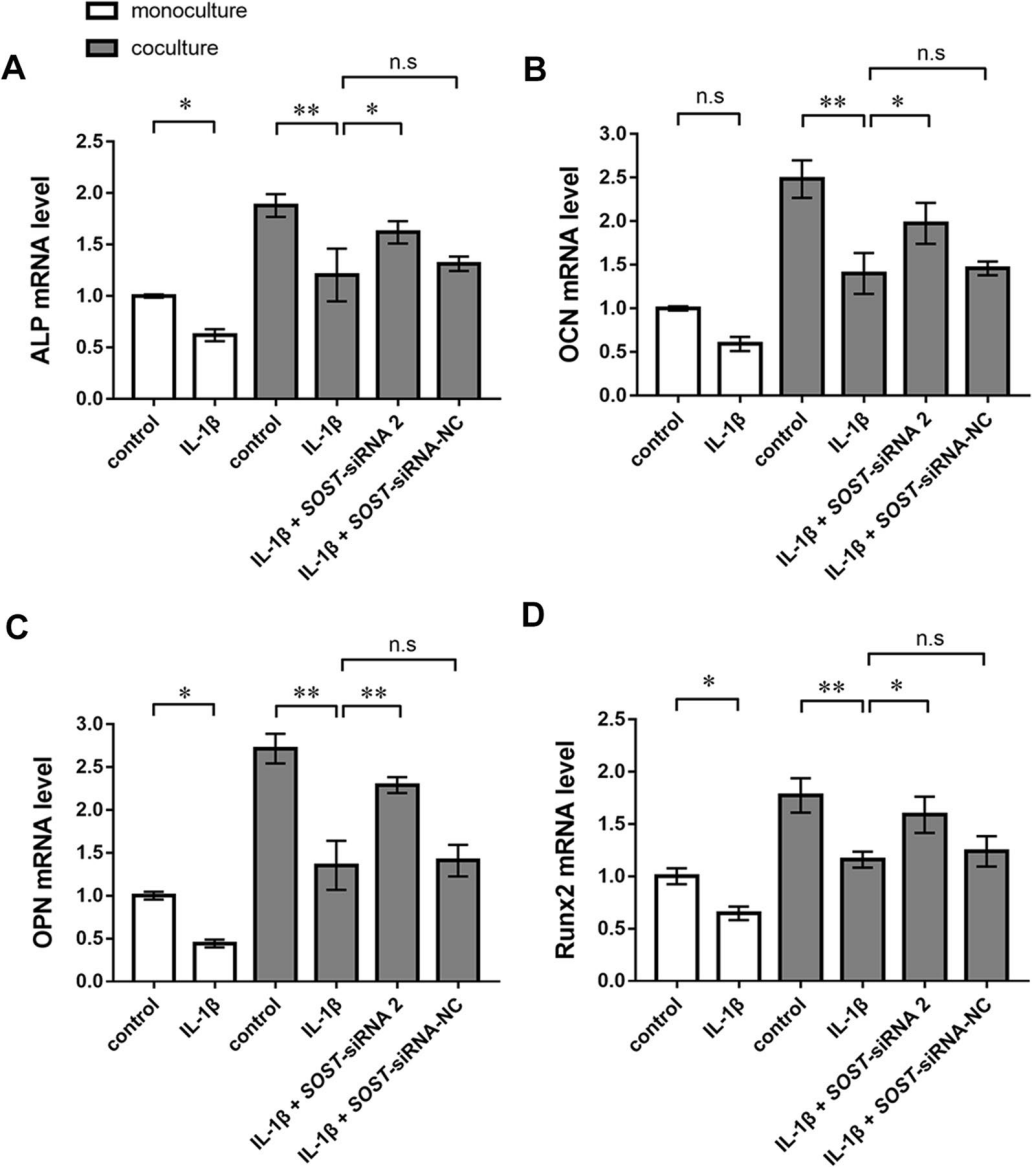
PCR results and ALP activity determination. The mRNA expression levels of ALP (**A**), OCN (**B**), OPN (**C**) and Runx2 (**D**) in MC3T3-E1 cells. IL-1β decreased the mRNA levels of ALP, OPN, OCN, and Runx2 while inhibiting sclerostin by Ocy454 cells increased the mRNA levels of ALP, OPN, OCN, and Runx2 under IL-1β-mediated inflammatory conditions. The experiments in vitro were performed independently 3 times. *p < 0.05 and **p < 0.01. n.s., not significant

To investigate whether MC3T3-E1 cells underwent osteogenic differentiation, we determined the mRNA and protein expression levels of ALP, OPN, OCN and Runx2 by PCR and western blotting. PCR revealed that exposure to 10 ng/ml IL-1β inhibited ALP, OPN and Runx2 mRNA expression in the MC3T3-E1 cell monoculture group. When cocultured with Ocy454 cells, exposure to 10 ng/ml IL-1β decreased the mRNA expression levels of ALP, OPN, OCN and Runx2 in MC3T3-E1 cells. Inhibiting the secretion of sclerostin from Ocy454 cells led to increased mRNA expression of ALP, OPN, OCN and Runx2 in MC3T3-E1 cells. There were no significant differences in the mRNA expression levels of ALP, OPN, OCN and Runx2 between the IL-1β group and the siRNA-negative control (siRNA-NC) group (Fig. 2A–D).

**Fig. 3:**
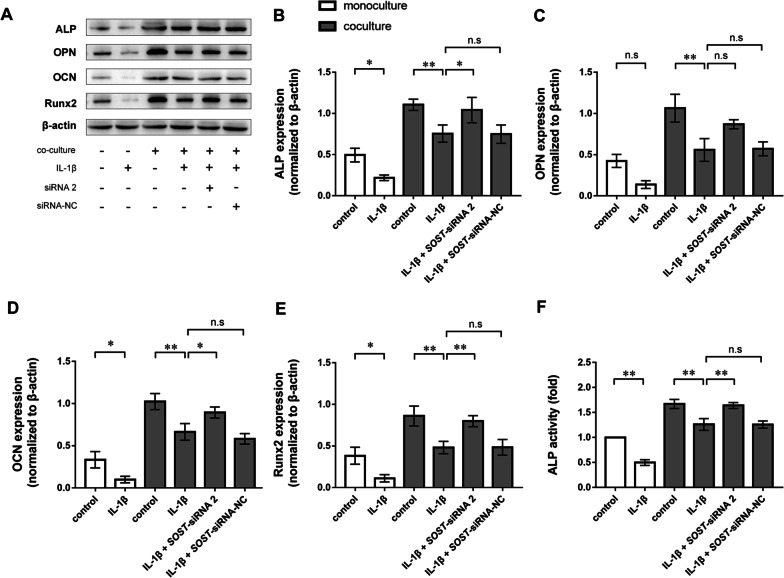
Western blotting of ALP, OPN, OCN, and Runx2 protein expression in MC3T3-E1 cells. **A** Representative western blots. Quantitative analysis of ALP (**B**), OPN (**C**), OCN (**D**), and Runx2 (**E**) protein expression levels in MC3T3-E1 cells. IL-1β suppressed the protein expression of ALP, OPN, OCN, and Runx2 while inhibiting sclerostin by Ocy454 cells enhanced the protein expression of ALP, OCN, and Runx2 under IL-1β-mediated inflammatory conditions. (F) ALP activity in MC3T3-E1 cells. The experiments in vitro were performed independently 3 times. *p < 0.05 and **p < 0.01. n.s., not significant

Western blotting revealed that exposure to 10 ng/ml IL-1β reduced ALP, OCN and Runx2 protein expression in the MC3T3-E1 cell monoculture group. The protein expression of ALP, OPN, OCN and Runx2 were downregulated in MC3T3-E1 cells cocultured with Ocy454 cells exposed to 10 ng/ml IL-1β. Inhibiting the secretion of sclerostin from Ocy454 cells enhanced protein expression of ALP, OCN and Runx2 in MC3T3-E1 cells. Transfecting Ocy454 cells with siRNA-NC did not affect ALP, OPN, OCN or Runx2 protein expression compared with the cocultured cells exposed to IL-1β (Fig. 3A–E).

**Fig. 4:**
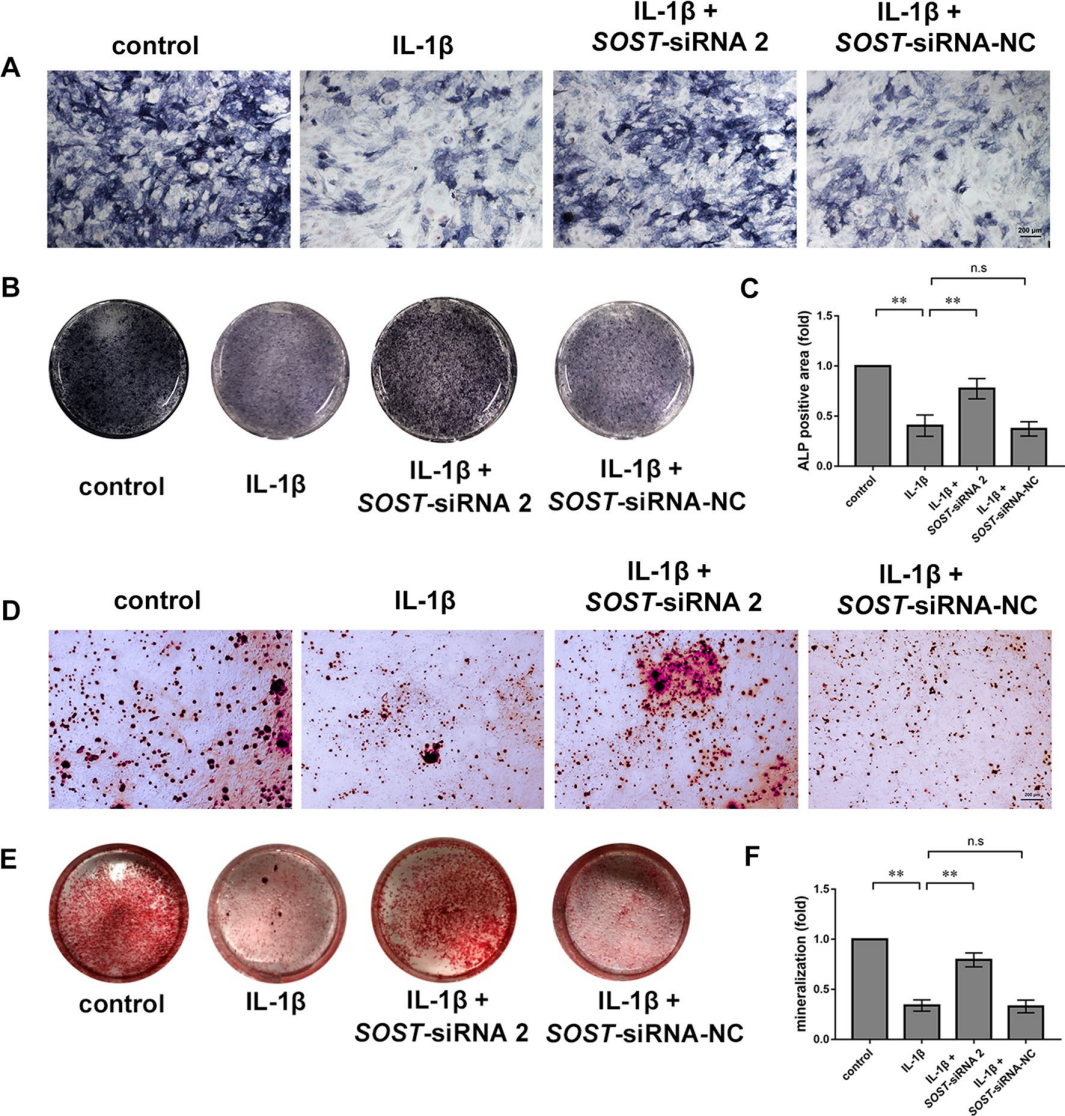
Alizarin red S staining and ALP staining of MC3T3-E1 cells. **A** Representative images of ALP staining. Scale bar, 200 µm. **B** Representative photographs of ALP stained wells. **C** Quantification of ALP positive area normalised to the control group. **D** Representative images of mineralised nodules stained with Alizarin red S. Scale bar, 200 µm. **E** Representative photographs of ARS stained wells. **F** Quantification of calcium deposition normalised to the control group. The experiments in vitro were performed independently 3 times. *p < 0.05 and **p < 0.01. n.s., not significant

Exposure to 10 ng/ml IL-1β significantly reduced ALP activity in MC3T3-E1 cells in monoculture and coculture conditions. Inhibiting the secretion of sclerostin from Ocy454 cells resulted in enhanced ALP activity in MC3T3-E1 cells. Transfection of Ocy454 cells with siRNA-NC did not affect ALP activity compared with the coculture group exposed to IL-1β (Fig. 3F). Ocy454 cells pretreated with IL-1β could also inhibited the ALP, OPN, and Runx2 protein expression levels in MC3T3-E1 cells. However, in the absence of IL-1β, inhibiting sclerostin expression in Ocy454 cells did not have significant impacts on the ALP, OPN, OCN, and Runx2 protein expression levels in MC3T3-E1 cells, indicating that the sclerostin secreted by osteocytes are insufficient to suppress osteogenic differentiation under physiological conditions (Additional file 3: Fig. S2). In addition, exposure to 10 ng/ml IL-1β significantly decreased ALP positive area of MC3T3-E1 cells. Inhibiting the secretion of sclerostin from Ocy454 cells increased ALP positive area of MC3T3-E1 cells (Fig. 4A–C).

### Inhibiting the secretion of sclerostin from Ocy454 promoted the mineralisation of MC3T3-E1 cells

Alizarin red S staining was used to evaluate the mineralisation of MC3T3-E1 cells in each coculture group. Of note, exposure to 10 ng/ml IL-1β significantly inhibited the mineralisation of MC3T3-E1 cells. By contrast, inhibiting the secretion of sclerostin from Ocy454 cells promoted the mineralisation of MC3T3-E1 cells. Transfecting Ocy454 with siRNA-NC did not affect the mineralisation of MC3T3-E1 cells (Fig. 4D–F).

### SOST knockout promoted bone healing in the rat spinal fusion model

**Fig. 5:**
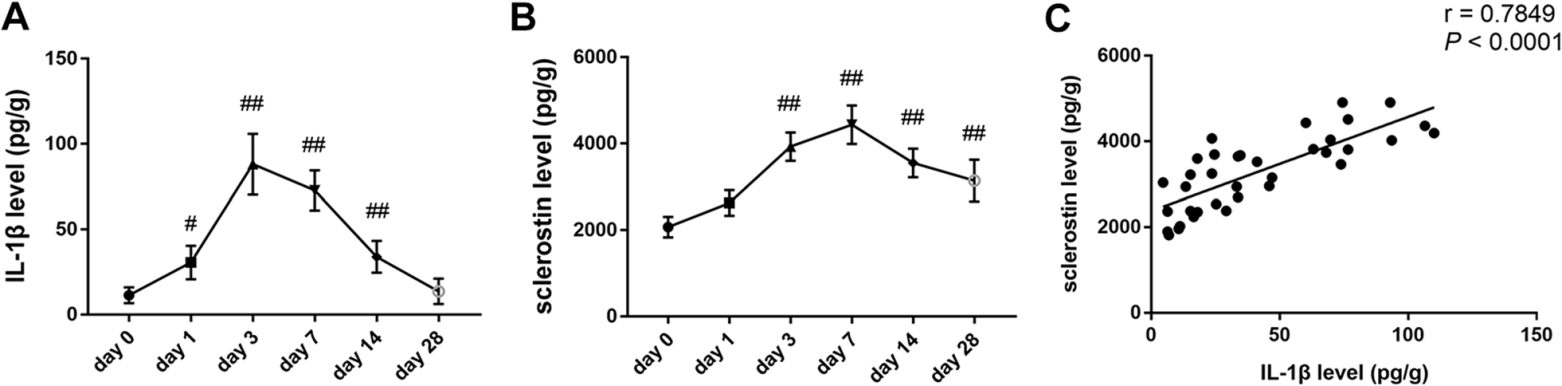
Evaluation of the correlation of IL-1β and sclerostin in vivo. Decorticated Transverse processes of at L4 and L5 in rats were harvested at day 0, 1, 3, 7, 14 and 28. IL-1β and sclerostin levels were assessed using ELISA. **A** IL-1β level in bone tissue. **B** Sclerostin level in bone tissue. **C** The correlation between IL-1β and sclerostin. There were 6 male rat in each group. #*p* < 0.05 and ##*p* < 0.01 versus day 0

In the in vivo study, bilateral posterolateral intertransverse process lumbar fusion surgery was performed to establish rat spinal fusion model. After operated, transverse processes L4 and L5 in rats were harvested. ELISA results showed IL-1β level was increased at day 1, 3, 7 and 14 and was maximal at day 3 (Fig. 5A). In addition, sclerostin level was increased at day 3, 7, 14 and 28. Sclerostin level reached a peak at day 7 (Fig. 5B). Correlation analyses showed that there was a statistically significant positive correlation between IL-1β and sclerostin levels in bone tissues (r = 0.7849, *p* < 0.0001) (Fig. 5C). These results can give us some hints that IL-1β is involved in the increased sclerostin at early stages of bone healing.

**Fig. 6:**
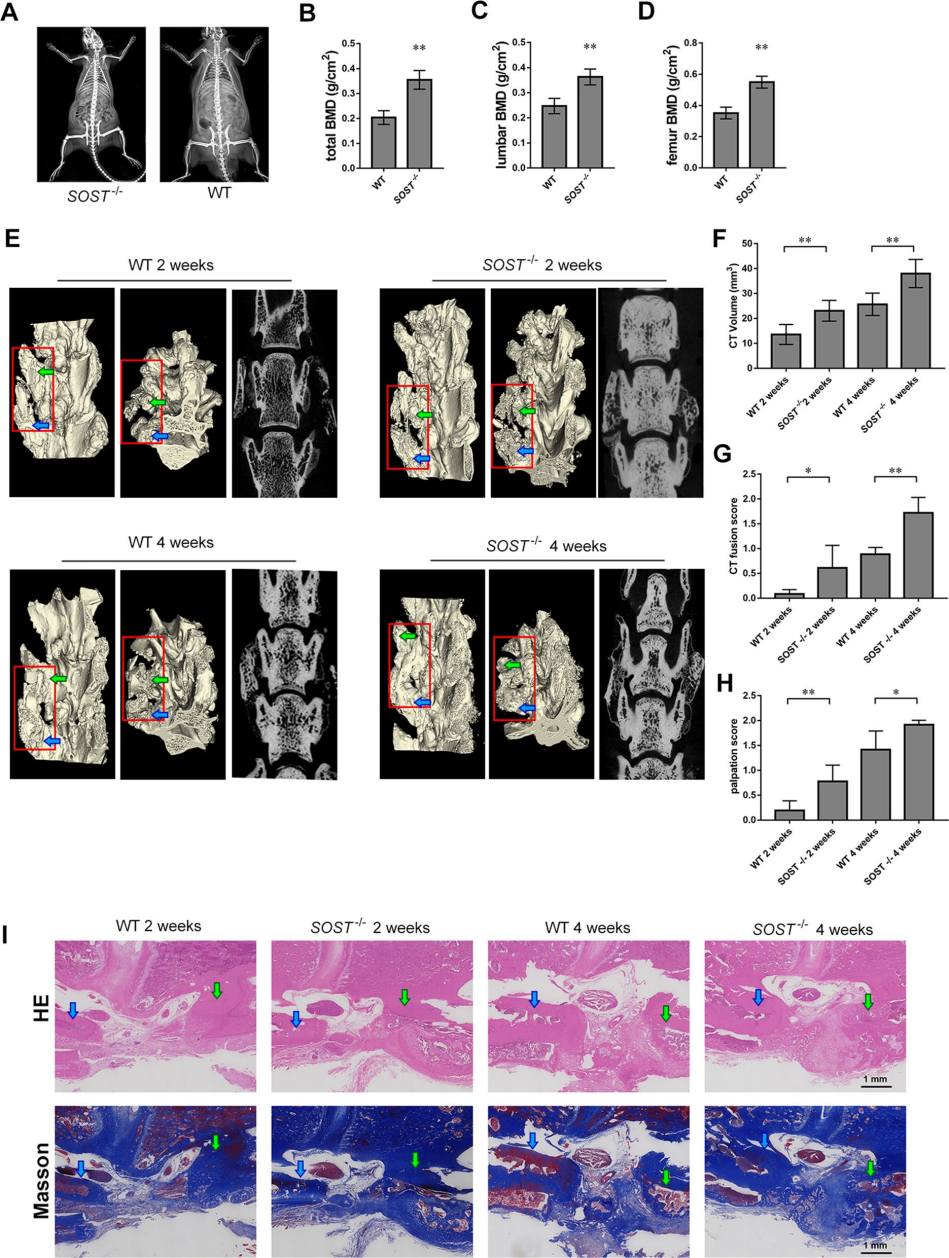
Evaluation of phenotype validation of SOST^−/−^ rats and assessment of spinal fusion. **A** BMD was measured by dual-energy X-ray absorptiometry. The total BMD (**B**), lumbar spine BMD (**C**) and femur BMD (**D**) in *SOST*^−/−^ rats were higher than those in WT rats, respectively. **E** Representative two-dimensional and three-dimensional reconstructed CT images. **F** Fusion-mass volume. **G** CT fusion scores. **H** Manual palpation scores. **I** Representative images of hematoxylin and eosin staining and Masson trichrome staining in the four experimental groups four experimental groups (2-week WT rats, 2-week *SOST*^−/−^rats, 4-week WT rats and 4-week *SOST*^−/−^ rats). Scale bar, 1 mm. Blue arrow: L4 transverse process. Green arrow: L5 transverse process. There were 6 male rat in each group. *p < 0.05 and **p < 0.01

Subsequently, we construct the *SOST*^−/−^ rat. Dual X-ray absorptiometry assessment showed the total BMD lumbar spine BMD and femur BMD in *SOST*^−/−^ rats were higher than those in WT rats, indicating sclerostin knock-out rats were characterized by increased bone density (Fig. 6A–D).

The mean palpation scores in the 2-week WT, 2-week *SOST*^−/−^, 4-week WT and 4-week *SOST*^−/−^ groups were 0.19 ± 0.19, 0.78 ± 0.33, 1.42 ± 0.38 and 1.92 ± 0.09, respectively (Fig. 6H). The palpation scores were significantly different between the WT and *SOST*^−/−^ groups at 2 weeks (*p* = 0.0068) and at 4 weeks (*p* < 0.0221). Representative two-dimensional and three-dimensional reconstructions of the micro-CT images are displayed in Fig. 6E. The mean CT fusion scores in the 2-week WT, 2-week *SOST*^−/−^, 4-week WT and 4-week *SOST*^−/−^ groups were 0.08 ± 0.09, 0.61 ± 0.46, 0.89 ± 0.14 and 1.72 ± 0.31, respectively (Fig. 6G). The fusion-mass volume (Fig. 6F) and CT fusion scores were significantly different between the WT groups and *SOST*^−/−^ groups at 2 weeks (*p* = 0.0224) and 4 weeks (*p* = 0.0004). H&E staining and Masson trichrome staining showed that the bone grafts were more obviously integrated with the surrounding bone in *SOST*^−/−^ groups compared with WT groups at 2 weeks and 4 weeks, respectively (Fig. 6I).

## Discussion

Spinal fusion is widely used in the treatment of spinal instability and spinal degenerative diseases. BMPs are used to promote the healing of lumbar fusion with degenerative disc disease, but they may cause complications such as local bone resorption, ectopic bone formation and edema [6–9]. Therefore, it is necessary to identify safe and effective osteogenic agents.

Sclerostin, which is secreted mainly by osteocytes, inhibits the canonical Wnt pathway and thus inhibits bone formation [17, 37]. Inflammation is an important factor affecting spinal fusion. IL-1β is induced to high activities in the early phase of bone healing process [23]. IL-1β-mediated inflammatory conditions may affect the development of spinal fusion by promoting sclerostin levels in the bone healing environment, which could be a potential therapeutic target to promote spinal fusion. We thus conducted the current experiment to determine the the connection between IL-1β and sclerostin in osteocytes and further explored whether inhibiting the secretion of sclerostin from osteocytes promotes spinal fusion at early stages of bone healing.

Our study showed IL-1β and sclerostin increased in bone from day 1 after modeling. IL-1β level showed positive correlations with sclerostin level in vivo. Previous study also showed that the sclerostin mRNA level in osteocytes in human trabecular bone chips was increased when incubated with IL-1β, but did not change when treated with TNF-a or IL-8 [19], suggesting IL-1β is an important inflammatory cytokine regulating sclerostin. We then explored the effects of IL-1β on osteocyte sclerostin expression and determined the most suitable concentration of IL-1β. The results of PCR and ELISA showed that 10 ng/ml IL-1β significantly enhanced the secretion of sclerostin from Ocy454 cells, partially accounting for the sharp increase of sclerostin in the early stage of bone healing process. A > sixfold increase in sclerostin expression was observed in transplanted iliac crest bone graft in the fusion bed 1 week after spinal fusion, suggesting that sharp increase in sclerostin during the bone graft healing process may have potential problems for the development of successful spinal fusion [20]. In the current study, we also found that sclerostin increased in bone from day 3 after modeling. IL-1β, a prominent cytokine released during spinal fusion, may be an explanation for the sharp risk of sclerostin [26, 38]. The effect of IL-1β on sclerostin at the level of mRNA and secretion was further verified in the current study.

A coculture system of osteocytes and osteoblasts was established to explore whether inhibiting IL-1β-induced secretion of sclerostin from Ocy454 cells promotes MC3T3-El cell osteogenic differentiation. We found that exposure to 10 ng/ml IL-1β inhibited the osteogenic differentiation of MC3T3-E1 cells. Exposure to 10 ng/ml IL-1β elicited similar effects of inhibiting osteogenic differentiation in the coculture groups. By contrast, inhibiting the secretion of sclerostin from Ocy454 cells promoted the osteogenic differentiation of MC3T3-E1 cells. These findings suggest that, in addition to the inhibitory effects of IL-1β itself on osteogenic differentiation, IL-1β-induced secretion of sclerostin from Ocy454 cells is an important regulator of MC3T3-E1 cell osteogenic differentiation. Furthermore, coculturing osteocytes with osteoblasts can promote osteogenic differentiation, as previously reported [39]. Alizarin red S staining at 21 days showed that exposure to IL-1β inhibited the mineralisation of osteoblasts in the coculture system. This effect was partly abolished by inhibiting the expression and secretion of sclerostin. IL-1β inhibited the osteogenic differentiation of osteoblasts in vitro by promoting the secretion of sclerostin from osteocytes. Inhibiting the secretion of sclerostin from Ocy454 cells promotes osteogenic differentiation of MC3T3-E1 cells under IL-1β-mediated inflammatory conditions in vitro.

A rodent spinal fusion model was established in *SOST*^−/−^ and WT SD rats to investigate whether the absence of sclerostin promotes bone graft healing in vivo. The extent of spinal graft fusion at 2 and 4 weeks after surgical induction was evaluated by manual palpation, radiography and histopathology. Of note, fusion-mass volume, palpation scores and CT fusion scores indicated that bone healing was significantly better in the *SOST*^−/−^ rats than in the WT rats at 2 and 4 weeks. These findings suggest that inhibiting the secretion of sclerostin from osteocytes promoted spinal fusion in vivo. Our results indicate that sclerostin is a potential therapeutic target for promoting spinal fusion at early stages. Due to an insufficient number of littermates, we used WT rats in the current study. In addition, to avoid the influence of any sexual endocrine disturbance, only male rats were used in this study.

It should be noted prior in vivo studies often used a time-point of 8 weeks. We did not use this time-point for several reasons: (1) IL-1β and sclerostin increase at early stages of bone healing, reaching peak levels at about 1–2 weeks and returning to normal by 4 weeks [23]; (2) in preliminary studies, we found that the bone grafts in the WT rats in the control group had healed normally by 8 weeks, making it difficult to compare them with the *SOST*^−/−^ rats using the available methods.

In conclusion, we have shown that IL-1β contributes to a rise in the level of sclerostin at early stages of bone healing. Inhibiting the secretion of sclerostin from osteocytes effectively promotes osteogenic differentiation and mineralisation of osteoblasts exposed to IL-1β in vitro and bone graft healing in vivo. Because we did not simulate long-term chronic inflammation in vivo in this study, future studies should assess this situation. Additionally, further experiments are required to investigate the effectiveness of exogenous anti-sclerostin drugs, such as romosozumab, on bone graft healing. Nevertheless, our findings highlight the potential of antisclerostin treatments for promoting spinal fusion at early stages.

## Conclusions

The present study showed IL-1β contributes to a rise in the level of sclerostin at early stages of bone healing. Inhibiting the secretion of sclerostin from osteocytes effectively promotes osteogenic differentiation and mineralisation of osteoblasts exposed to IL-1β in vitro and bone graft healing in vivo. The results suggest that antisclerostin treatment has the potential to effectively promote spinal fusion at early stages.

## Supplementary Information

**Additional file 1.** The construction strategy for SOST knockout rat via CRISPR/Cas 9 system.

**Additional file 2: Fig. S1.** Ocy454 cells were transfected with siRNA to inhibit the secretion of sclerostin. **A**, **B** Western blotting of sclerostin protein expression in Ocy454 cells at 48 h after transfection with one of three SOST-siRNAs. **C** Sclerostin mRNA levels in Ocy454 cells at 48 h after transfection with one of three SOST-siRNAs. **D** ELISA of sclerostin protein levels in the supernatant. SOST-siRNA2 had the greatest inhibitory effect on the secretion of sclerostin from Ocy454 cells. There were 6 male rats in each group. *p < 0.05 and **p < 0.01 vs. control cells

**Additional file 3: Fig. S2.** Western blotting of ALP, OPN, OCN, and Runx2 protein expression in MC3T3-E1 cells cocultured with Ocy454 cells in medium without IL-1β. In the IL-1β pretreatment group, Ocy454 cells were pretreated with IL-1β for 24 h and the cocultured with MC3T3-E1 cells in fresh medium without IL-1β. **A** Representative western blots. Quantitative analysis of ALP (**B**), OPN (**C**), OCN (**D**), and Runx2 (**E**) protein expression levels in MC3T3-E1 cells. Inhibiting sclerostin expression in Ocy454 cells did not have significant impacts on the ALP, OPN, OCN, and Runx2 protein expression levels in MC3T3-E1 cells. After IL-1β pretreated, Ocy454 cells significantly inhibited the ALP, OPN, and Runx2 protein expression levels in MC3T3-E1 cells. The experiments in vitro were performed independently 3 times. *p < 0.05. n.s., not significant.

## Data Availability

The datasets used and/or analyzed during the current study are available from the corresponding author on reasonable request.

## Abbreviations

IL-1β: Interleukin-1β
TNFα: Tumour necrosis factor-α
BMP: Bone morphogenetic protein
α-MEM: α-Minimum essential medium
ALP: Alkaline phosphatase
OCN: Osteocalcin
OPN: Osteopontin
Runx2: Runt-related transcription factor 2
siRNA: Small-interfering RNA
PCR: Polymerase chain reaction
GAPDH: Glyceraldehyde 3-phosphate dehydrogenase
ELISA: Enzyme-linked immunosorbent assay
