# Supplementary material for: IL-1β contributes to the secretion of sclerostin by osteocytes and targeting sclerostin promotes spinal fusion at early stages
Source: J Orthop Surg Res. 2023 Mar 3;18:162. doi: 10.1186/s13018-023-03657-0 (PMC9983224; doi:10.1186/s13018-023-03657-0)
Supplement: Supplementary file 1 — Additional file 1. The construction strategy for SOST knockout rat via CRISPR/Cas 9 system. [file 13018_2023_3657_MOESM1_ESM.docx]

We made SOST knockout rat via CRISPR/Cas 9 system. Cas9 mRNA and sgRNA were con-injected into zygotes. sgRNA direct Cas9 endonuclease cleavage in intron 1-2 and downstream of exon2, and create a DSB (double-strand break). Such breaks will be repaired by non-homologous end joining (NHEJ) and result in disruption of SOST gene. The gene sequence and sgRNA sequences are as follows：

**SOST -201 (ENSRNOT00000028238.2)**


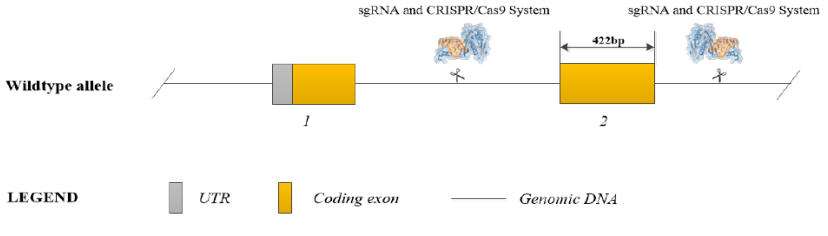


**Sequences**

exon1:

ACCGAGTGCCCTCCTCCTTCTGGCACCATGCAGCTCTCACTAGCCCCTTGCCTTGCCTGCCTGCTTGTACATGCAGCCTTCGTTGCTGTGGAGAGCCAGGGGTGGCAAGCCTTCAAGAATGATGCCACAGAAATCATCCCGGGACTCAGAGAGTACCCAGAGCCTCCTCAGGAACTAGAGAACAACCAGACCATGAACCGGGCCGAGAACGGAGGCAGACCCCCCCACCATCCTTATGACACCAAAG

intron 1-2

GTATGGGATGGAAAAGACAGTTGTTAGTTAGCACGGAGACCTGGGTGGTGACTGGGGTGGCTTTTAGAATCTTCTTTGGAGGTTCGTGTGGGTGGTTAAGCCTCTGCCACACCAGGGAAGGGACAGACTCACCTGGAAAAATTCTAGCTCAGTGTTAGAATCTGGAGGACAGGGTTGGGGGGCTGGCGGTGAGCACCCAAGGCAAGGTGGAGGGTGAGGTCAGCCAAAGCTGGCATTAACACAGGAATGGGCTTGAAGGGTGGTTCAGAGACTCTCCTGAAGGTGAGGACCAGTGGGGGAGGTGACAAGGTGAGGTTGATAGCACCTGCTGGATGCCAGATGCCGTGGCCATTACTGTATCCCACATGACCACCCCATGAGGTAAAGAAGGCCTTAGCTTGAAGGTGAAGAAACCGAGGCTCCTGAGATTAAAGTCACTTGGGGGTAAGAAGAGCTGAGACTGGAAGTTGGTTTGATCCAGATGCAAGGCAACCCTAGATTGGGTTTGGGTGGGAACCTGAAGCCAGGAAGAATCCCTTTAGTCCCCCTTGTCCATGATCTACCCAATGGGCCCAGTGGGCTAGCGTTAAAGAAACAACAGGGTTTGTAGGTACGGTTTTAGCATGTGACCTGAGGGGCAGTTGAGTGAAGCATCCCCTGTATGGGCACAGGTGGCATCATCTGCCCTGAGCTTATGCCCTGGCCCCAGTTTTGCCTCATTCCTGAGGACAGCAGGGCCTGTGGTAGCCTGCACAGAGAGATGCCTGGGGGATCAAGCACAGAGCTGGCAGGAATGAAATGGGTGGGGTGGCAGCTAGAGTGACACTCCACAGCGAGGACCTTGCTGGTCACCATTTGAGTAGGAGGAAAGGTCATTTTCCAGGTTGCCACCACACTCTGTCCCTCCCGTCTCCTAGCCAGTAAGGAGTTGGGGAGGGGGGGA

AGGGCCACCCCAAAGGAGCACATGCAATGCAGTCACGTTGTGCAGAGGAAGTGCTTGACCTAAGGGCACTATTCTTGGAAAGCCCCAAAACGAGTCCTTCCCTGGGCACACAGGCCGCCCCCACATACCACCTCTGCAACGGCGAGTAAATTAAGCCAGCCACAGGCGGGTGGCAGGCCTACACCTCCCCTGCTGTGCCTCCTCTCTGGGGCAAAGGTGGGTCCTGGTCTCTGCCCCTCTGGCTTTGGTCCTGGCCCCCCTTTTTTTTTTAAATCCTTTTATGTCATATTGGTTCTGACACCATGAAATCTTTGGAGGTGGACAGGACCTGCATGTGGATTCGTTCATTTAAGACCTCCCATTCATCTAAGCTCACGGTAGGAGATAGAGCCTGGCCATGTATAAGAGATGAGGCCAGGCATCAGCCCAGAGGATATAACCGGGCATCCAACCCAATCTCCTTCCACAGAGAACAGACCCCAAGTCAGATCCAGTCACCCTTGAGTTACCAGCTCAAGGTACACAGAATGAGAGAATCTGGTGCTCAACGAATGCCTATGGTAGCAGAGGCCGTAGGTTTTGGGTCAGAACTCTGACCTGATGCTAAGCAAGTATCCAGGAAAAAGCAATAGGGCCCTCTATCCTGCCCCACCCCCACCCATCCCCAACCTGGGGCCCTATAACAAATCACTTTCACCCTTGAGGGAACCAGAGAATTCTGGCAGCCCAGTCCTGCCTTGGGGGCAAGTTCTTTTCTCAGCCCGGACCCGTGATAATGAGGGGGTTGGACACGCTGCCTTTGGTCGCTTTCAAGTCTAATGAATTCTTATCCCCACCACCTGCCCTTCTACCCGCTCCTCCACAGCAGCTGTCCTGATTTATTACCTTCAATTAACCTCCACTTCTTTCCCATCTCCTGGGACCCCGCCCCTGCCCCAGTAGCTGGTAAAGTGTAGGGGGGACCAGAGCAAGCCAGGTGTGGCTAGAGGCTGGCTACCAGGCAGGGCTGGGGATAAGAGTGCTTAGTTAGTGCGCAGGAAGCCTTGGCATCTCTAGTACCAGGGAGATGGAGATAGGCGAAAGAAATGAACTCAAGACCATCCTCAACAACACTGCCCGGTGTAATGGTGGATGAAGTCCTCGAATCCTGGCGACCTATTTTACAGAGGTGGGGAAGAGCAACTTTAACTGCCCTGCCCACAGATCACACAGGAAGTGAATGATAGAGCGCCAGTGTTTCATCCCGGGAAGGGGGGGCGGACCAGGGCGGGAATCTCCCTGCTTTCCGAAAGCTCCAAAAGTACCCGGTGTCTCCTTCCTATAATCCACGCAGATTCGAAAACGCAGGGCAGGTTTGGAAAATGAGGGAGGGGTGGAAAGAGCAGTCCAGCCTGGCCTAGGCTGCAGCCCCTCACGTATCCCTCTCCCCGCAG

exon2:

ACGTGTCCGAGTACAGCTGCCGCGAGCTGCACTACACCCGCTTCGTGACCGACGGCCCGTGCCGCAGTGCCAAGCCGGTCACCGAGTTGGTGTGCTCGGGCCAGTGCGGCCCCGCGCGGCTGCTGCCCAACGCCATCGGGCGCGTGAAGTGGTGGCGCCCGAACGGACCCGACTTCCGCTGCATCCCGGATCGCTACCGCGCGCAGCGGGTGCAGCTGCTGTGCCCCGGCGGCGCGGCGCCGCGCTCGCGCAAGGTGCGTCTGGTGGCCTCGTGCAAGTGCAAGCGCCTCACCCGCTTCCACAACCAGTCGGAGCTCAAGGACTTCGGACCTGAGACCGCGCGGCCGCAGAAGGGTCGCAAGCCGCGGCCCCGCGCCCGGGGAGCCAAAGCCAACCAGGCGGAGCTGGAGAACGCCTACTAG

Table 2 sgRNA Sequences

| Name | Sequences | PAM |
| --- | --- | --- |
| SOST-5s1 | AAGGTCGGTGTGAACGGATT | TGG |
| SOST-5s2 | CGTGCCTCATCTGCCTACTTGT | TGG |
| SOST-3s1 | ACGGCGTCCATGAGCAGAACTA | GGG |
| SOST-3s2 | CCAAGCAGGAGGGCAATAAGGT | AGG |
