# Supplementary figures and images for: IL-1β contributes to the secretion of sclerostin by osteocytes and targeting sclerostin promotes spinal fusion at early stages
Source: J Orthop Surg Res. 2023 Mar 3;18:162. doi: 10.1186/s13018-023-03657-0 (PMC9983224; doi:10.1186/s13018-023-03657-0)

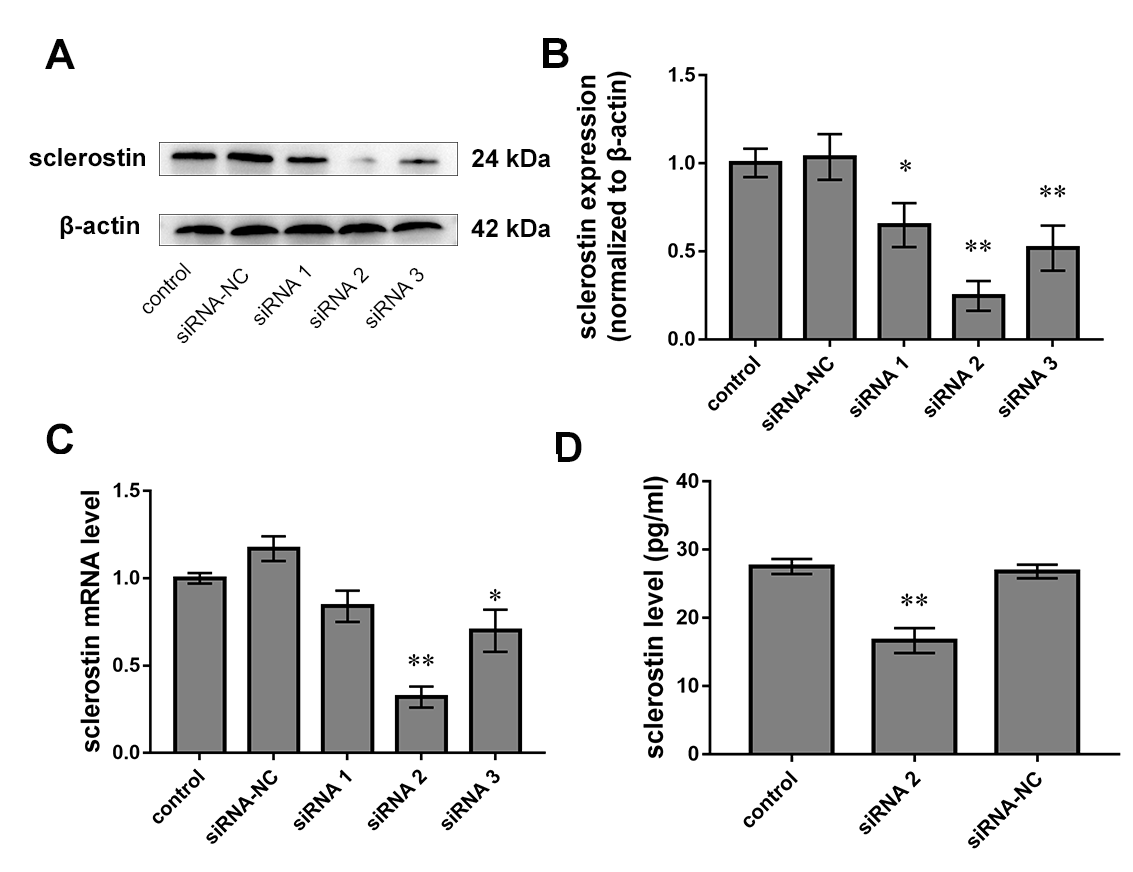

Supplement: Supplementary file 2 — Additional file 2: Fig. S1. Ocy454 cells were transfected with siRNA to inhibit the secretion of sclerostin. A, B Western blotting of sclerostin protein expression in Ocy454 cells at 48 h after transfection with one of three SOST-siRNAs. C Sclerostin mRNA levels in Ocy454 cells at 48 h after transfection with one of three SOST-siRNAs. D ELISA of sclerostin protein levels in the supernatant. SOST-siRNA2 had the greatest inhibitory effect on the secretion of sclerostin from Ocy454 cells. There were 6 male rats in each group. *p < 0.05 and **p < 0.01 vs. control cells [file 13018_2023_3657_MOESM2_ESM.tif]

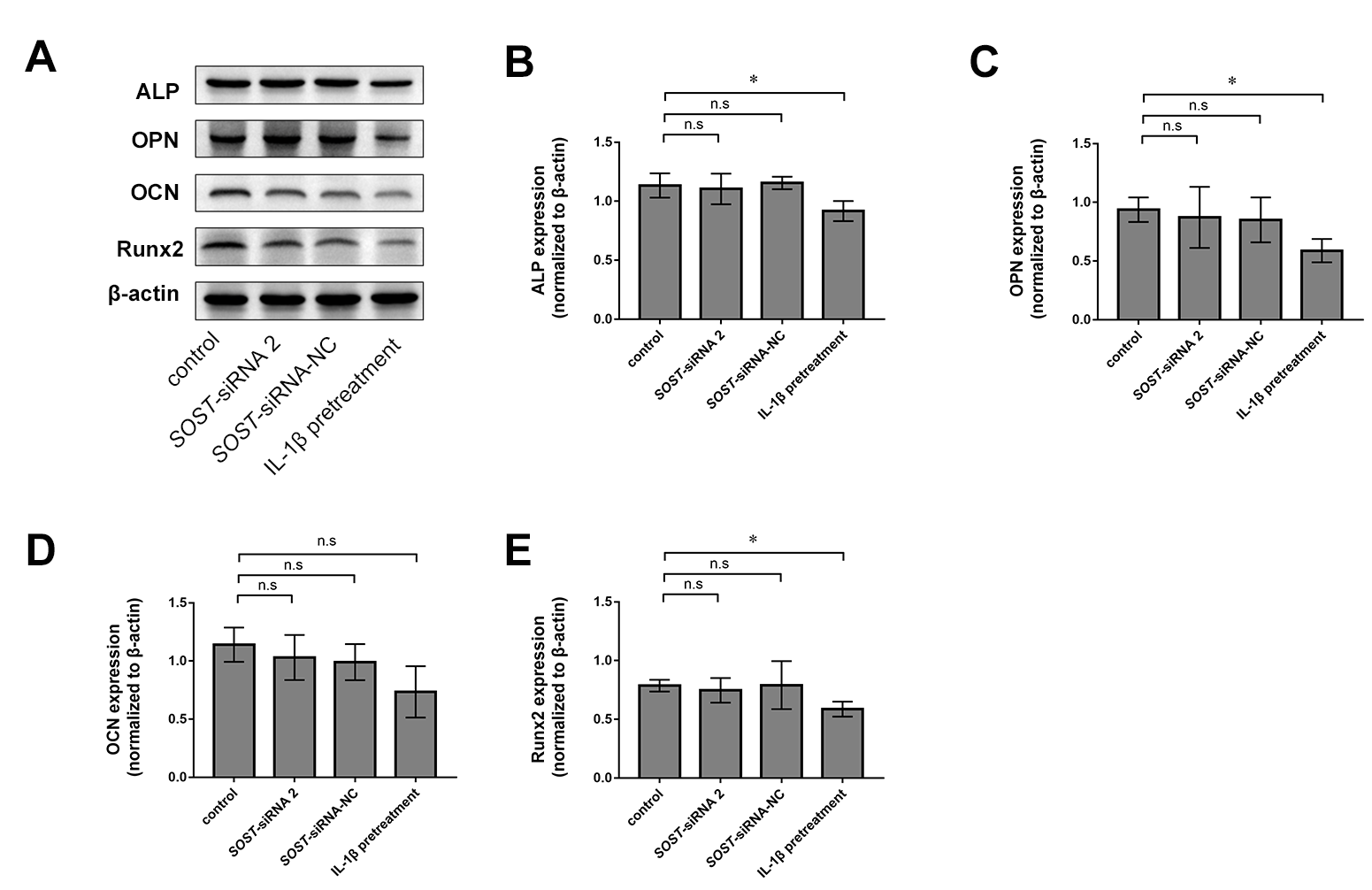

Supplement: Supplementary file 3 — Additional file 3: Fig. S2. Western blotting of ALP, OPN, OCN, and Runx2 protein expression in MC3T3-E1 cells cocultured with Ocy454 cells in medium without IL-1β. In the IL-1β pretreatment group, Ocy454 cells were pretreated with IL-1β for 24 h and the cocultured with MC3T3-E1 cells in fresh medium without IL-1β. A Representative western blots. Quantitative analysis of ALP (B), OPN (C), OCN (D), and Runx2 (E) protein expression levels in MC3T3-E1 cells. Inhibiting sclerostin expression in Ocy454 cells did not have significant impacts on the ALP, OPN, OCN, and Runx2 protein expression levels in MC3T3-E1 cells. After IL-1β pretreated, Ocy454 cells significantly inhibited the ALP, OPN, and Runx2 protein expression levels in MC3T3-E1 cells. The experiments in vitro were performed independently 3 times. *p < 0.05. n.s., not significant. [file 13018_2023_3657_MOESM3_ESM.tif]
